# The Impact of Prophylactic Dexamethasone on Nausea and Vomiting after Thyroidectomy: A Systematic Review and Meta-Analysis

**DOI:** 10.1371/journal.pone.0109582

**Published:** 2014-10-16

**Authors:** Zhenhong Zou, Yuming Jiang, Mingjia Xiao, Ruiyao Zhou

**Affiliations:** 1 Department of General Surgery, Nanfang Hospital, Southern Medical University, Guangzhou City, Guangdong Province, China; 2 Department of Hepatobiliary Surgery, Wuxi People’s Hospital of Nanjing Medical University, Wuxi, Jiangsu Province, China; 3 Department of General Surgery, The Third Affiliated Hospital of Wenzhou Medical University, Ruian City, Zhejiang Province, China; University of Michigan, United States of America

## Abstract

**Background:**

We carried out a systematic review and meta-analysis to evaluate the impact of prophylactic dexamethasone on post-operative nausea and vomiting (PONV), post-operative pain, and complications in patients undergoing thyroidectomy.

**Methods:**

We searched Pubmed, Embase, and Cochrane Library databases for randomized controlled trials (RCTs) that evaluated the prophylactic effect of dexamethasone versus placebo with or without other antiemetics for PONV in patients undergoing thyroidectomy. Meta-analyses were performed using RevMan 5.0 software.

**Results:**

Thirteen RCTs that considered high quality evidence including 2,180 patients were analyzed. The meta-analysis demonstrated a significant decrease in the incidence of PONV (RR 0.52, 95% CI 0.43 to 0.63, *P*<0.00001), the need for rescue anti-emetics (RR 0.42, 95% CI 0.30 to 0.57, *P*<0.00001), post-operative pain scores (WMD –1.17, 95% CI –1.91 to –0.44, *P* = 0.002), and the need for rescue analgesics (RR 0.65, 95% CI 0.50–0.83, *P* = 0.0008) in patients receiving dexamethasone compared to placebo, with or without concomitant antiemetics. Dexamethasone 8–10****mg had a significantly greater effect for reducing the incidence of PONV than dexamethasone 1.25–5****mg. Dexamethasone was as effective as other anti-emetics for reducing PONV (RR 1.25, 95% CI 0.86–1.81, *P* = 0.24). A significantly higher level of blood glucose during the immediate post-operative period in patients receiving dexamethasone compared to controls was the only adverse event.

**Conclusions:**

Prophylactic dexamethasone 8–10****mg administered intravenously before induction of anesthesia should be recommended as a safe and effective strategy for reducing the incidence of PONV, the need for rescue anti-emetics, post-operative pain, and the need for rescue analgesia in thyroidectomy patients, except those that are pregnant, have diabetes mellitus, hyperglycemia, or contraindications for dexamethasone. More high quality trials are warranted to define the benefits and risks of prophylactic dexamethasone in potential patients with a high risk for PONV.

## Introduction

Post-operative nausea and vomiting (PONV) is a common and distressing complication associated with surgery. The overall incidence of PONV ranges from 20 to 30% in general surgery and up to 80% in high-risk surgical patients when no prophylactic anti-emetic is given [1], [2]. For patients undergoing thyroidectomy, PONV is a risk factor for post-operative bleeding [3], [4], and prophylactic anti-emetics may be beneficial.

Previous studies have shown that prophylactic dexamethasone has anti-emetic and analgesic effects. Glucocorticoids are anti-inflammatory and immunosuppressive agents, and dexamethasone may exert its therapeutic actions through central inhibition of prostaglandin synthesis, by decreasing serotonin turnover in the central nervous system, and by influencing the systemic inflammatory response in favor of anti-inflammatory mediators [5]–[9].

A systematic review demonstrated that prophylactic dexamethasone was safe and effective for reducing the incidence of PONV and post-operative pain in patients undergoing laparoscopic cholecystectomy compared to placebo [10]. In patients undergoing thyroidectomy, a previous meta-analysis demonstrated a significant reduction of PONV in patients treated with a single dose of dexamethasone versus placebo [11]. However, the relatively small sample size included in this review precluded the authors from drawing definitive conclusions, and the optimal dose and timing of dexamethasone administration, and efficacy of combining dexamethasone with other anti-emetics, remains unclear.

The objective of the current study was to confirm, and continue to investigate the impact of prophylactic corticosteroid administration on PONV, post-operative pain, and complications following thyroidectomy.

## Methods

This systematic review and meta-analysis is reported in accordance with the recommendations of the PRISMA statement [12].

### 2.1 Outcome measures

#### 2.1.1 Primary outcome measure

1. Incidence of PONV during the immediate 24****h post-operative period, dichotomized as no nausea versus others; this was evaluated according to a 3-point ordinal scale: no nausea; nausea; retching and/or vomiting

#### 2.1.2 Secondary outcome measures

1. Post-operative pain scores
2. Need for rescue anti-emetic or analgesic agent(s)
3. Incidence of steroid-related complications, including hyperglycemia, wound infection, delayed wound healing, headaches, dizziness, facial flushing, constipation, and abdominal pain

### 2.2 Data collection and analysis

#### 2.2.1 Searches

We searched PubMed, Embase, and Cochrane Library databases from their inception to October 1, 2013 using Cochrane Highly Sensitive Search Strategies to identify randomized controlled trials (RCTs) for potential inclusion in our review [13]. We used the following MeSH terms and keywords: thyroid surgery OR thyroidectomy AND corticosteroid, glucocorticoid, steroid, OR dexamethasone. The search strategy is summarized in Table S1. Authors’ names were entered as search terms in the PubMed database to check for additional studies. Trials were also identified using the “related articles” function in PubMed. We hand-searched reference lists from articles identified by the electronic search and from previous meta-analyses. This process was performed iteratively until no additional articles could be identified.

#### 2.2.2 Inclusion and Exclusion Criteria

We included RCTs that: evaluated the prophylactic effect of dexamethasone versus placebo without other anti-emetics, dexamethasone versus placebo plus concomitant administration of a different anti-emetic, dexamethasone versus a different anti-emetic, and comparisons using different doses of dexamethasone for PONV in patients undergoing thyroidectomy. The included trials reported at least one of our outcome measures, and clearly reported patient inclusion and exclusion criteria, anesthetic technique, protocols for administration of the experimental drugs, and a definition and evaluation of nausea and vomiting. Studies were excluded if they were not RCTs, included patients who were undergoing other surgical procedures concomitantly, reported insufficient data, or were duplicate studies.

#### 2.2.3 Selection of studies

Two reviewers (ZH Zou and YM Jiang) independently examined titles and abstracts to select eligible RCTs. We removed records that were ongoing or unpublished studies, or were published as abstracts or conference proceedings. Where datasets were overlapping or duplicated, only the most recent information was included. We retrieved the full text of potentially relevant studies. Two reviewers (ZH Zou and YM Jiang) independently examined the full text records to determine which studies met the inclusion criteria. We resolved disagreements about selection of studies by discussion and consensus.

#### 2.2.4 Data extraction and management

Two reviewers (ZH Zou and YM Jiang) independently extracted data from eligible RCTs including details describing study population, interventions, and outcomes. We resolved disagreements about data extraction by discussion and consensus.

#### 2.2.5 Assessment of quality of evidence in included studies

Two reviewers (ZH Zou and YM Jiang) independently assessed RCT quality and risk of bias using tools provided by the Cochrane Collaboration [14]. The reviewers examined six domains including sequence generation, allocation concealment, double-blind evaluation (blinding), complete outcome data, selective outcome reporting, and baseline comparability of groups. The risk of bias was categorized as low, high, or unclear. RCTs with high risk of bias in at least three of six domains were not included in the meta-analysis. Baseline comparability of groups was assessed using seven matching criteria: age, sex, history of motion sickness, previous post-operative emesis, anesthetic technique, operation type (partial or total thyroidectomy), and duration of surgery. Baseline incomparability was defined as non-matching in at least three of the seven criteria. We resolved disagreements about quality of evidence by discussion and consensus.

#### 2.2.6 Statistical analysis

Statistical analyses were performed using RevMan (ver. 5.0; The Cochrane Collaboration, Oxford, UK) software and STATA (ver. 11.2; STATA Corporation, College Station, TX, USA) software. Weighted mean differences (WMDs) with 95% confidence intervals (CIs) were calculated for continuous variables, and risk ratios (RRs) with 95% CIs were calculated for dichotomous variables. A random-effects model was used to pool studies with significant heterogeneity, as determined by the chi-squared test (*P*≤0.10) and the inconsistency index (*I^2^*≥50%). Potential sources of statistical heterogeneity were explored by carrying out subgroup and sensitivity analyses. Subgroup analyses were performed by stratifying patients according to dose of corticosteroid and timing of dexamethasone administration; sensitivity analyses explored the impact of excluding outlying results. The presence of publication bias was comprehensively assessed using Begg’s funnel plot and Begg’s rank correlation test of asymmetry. Publication bias was thought to be present when the continuity-corrected *Pr*>|*z*| value was ≤0.1 [15]. The GRADE system was used to summarize the overall quality of evidence [16], [17].

## Results

### 3.1 Trial identification

**Figure 1:**
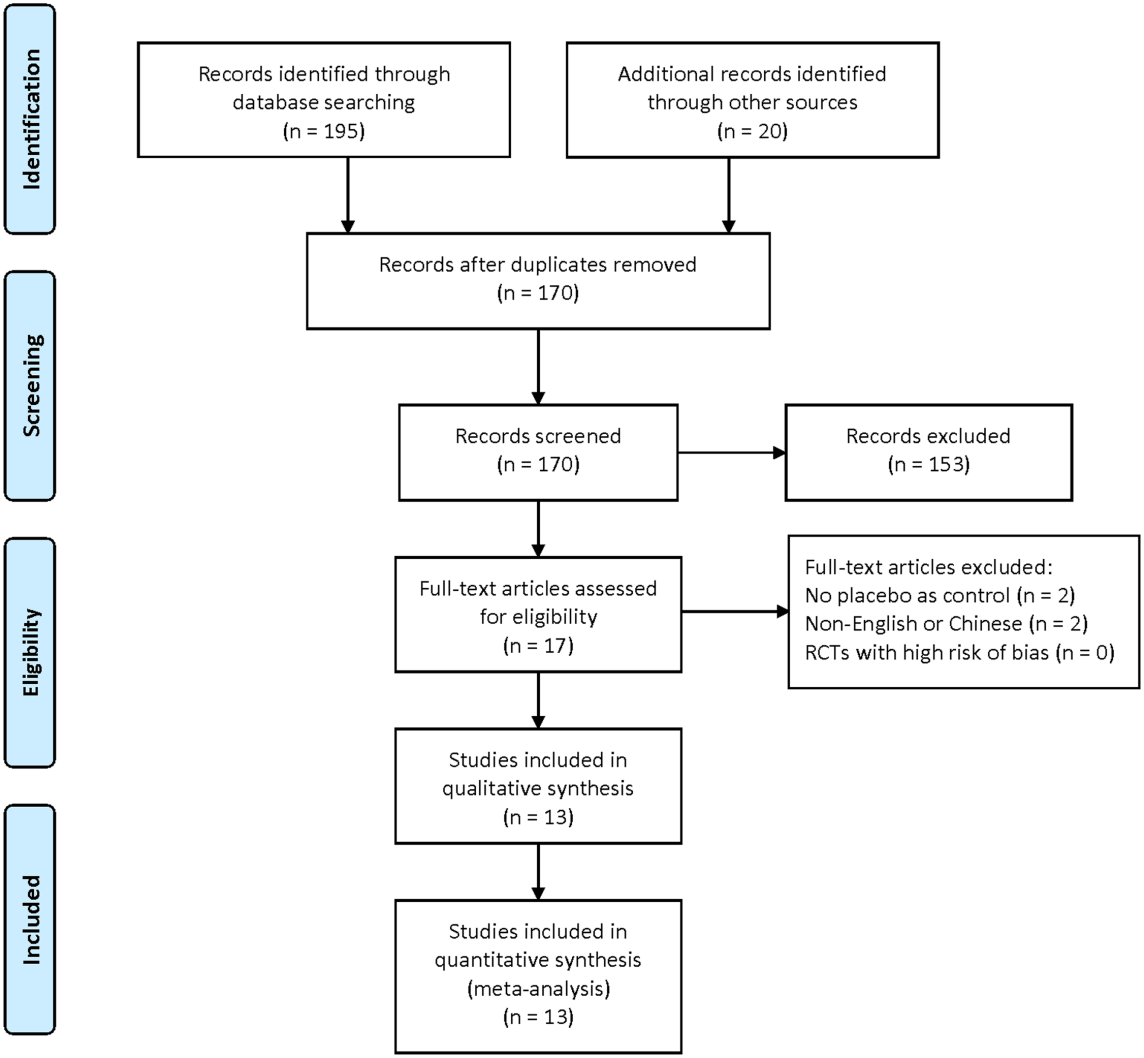
Flow chart for selecting the trials. On the basis of the search strategy, 195 articles were identified by the initial search, and 17 required further assessment. Finally, 13 articles were included in this review.

The searches identified 195 articles. We screened titles and abstracts, and 17 were identified as potentially eligible for inclusion. We retrieved the full text articles. After analyzing the full text articles, 4 studies were excluded and 13 RCTs [18]–[30] were found eligible for inclusion according to our criteria for considering studies in this review (Fig. 1).

### 3.2 Characteristics of included studies

The characteristics of the included studies are shown in Table 1. The 13 eligible RCTs included 2,180 patients who underwent general anesthesia for thyroidectomy. The majority of RCTs included patients classified as American Society of Anesthesiologists (ASA) class I or II. Exclusion criteria were: pregnant women, patients with insulin-dependent diabetes mellitus, obesity, and patients with a high risk for PONV. Dexamethasone was administered intravenously in a single or combination dose ranging from 1.25–18****mg. Timing of administration varied from 90****minutes before skin incision to the end of surgery. Controls included placebo, droperidol, granisetron, ondansetron, tropisetron or a combination of these medications. Confounders such as anesthetic technique and rescue analgesics and anti-emetics were standardized within studies (Table 2). Risk of bias was low across all RCTs (Table 3).

**Table 1:** Characteristics of trials included in the meta-analysis.

Study	Samplesize	Interventions	Studies divided
Wang1999 [18]	120	D 10 mg vs. Droperidol1.25****mg vs. Placebo, all IVat 1****minute before induction	Wang 1999 D 10: D 10 mg vs.Placebo; Wang 1999: D 10 mg vs.Droperidol 1.25 mg
Wang2000 [19]	225	D 10 mg vs. D 5 mg vs. D2.5 mg vs. D 1.25 mg vs. Placebo,all IV immediately after induction	Wang 2000 D 10: D 10 mg vs.Placebo; Wang 2000 D 5: D 5 mg vs.Placebo; Wang 2000 D 2.5: D 2.5 mg vs.Placebo; Wang 2000 D 1.25: D 1.25 mg vs. Placebo
Lee2001 [20]	135	D 8 mg vs. D 5 mg vs. Placebo,all IV before anesthesia	Lee 2001 D 8: D 10 mg vs.Placebo; Lee 2001 D 5: D 5 mg vs. Placebo
Fujji2007 [21]	75	D 8 mg vs. D 4 mg vs. Placebo,all IV at the end of surgery	Fujji 2007 D8: D 8 mg vs.Placebo; Fujji 2007 D4: D 4 mg vs. Placebo
Worni2008 [22]	70	D 8 mg vs. Placebo, both IVat 45****minutes before anesthesia	Worni 2008 D8: D 8 mg vs. Placebo
Feroci2010 [23]	102	D 8 mg vs. Placebo, both IVat 20****minutes before induction	Feroci 2010 D8: D 8 mg vs. Placebo
Doksrod2012 [24]	120	D 0.3 mg/kg vs. D 0.15 mg/kg vs.Placebo, all IV within10 min after induction	Doksrod 2012 D18: D 0.3 mg/kg vs. Placebo;Doksrod 2012 D9: D 0.15 mg/kg vs. Placebo
Song2013 [25]	123	D 10 mg vs. Ramosetron0.3 mg vs. Placebo, both IVimmediately after anesthesia	Song 2013 D 10: D10: D 10 mg vs.Placebo; Song 2013: D 10 mg vs.Ramosetron 0.3 mg
Barros2013 [26]	40	D 4 mg vs. Placebo, bothIV immediately after induction	Barros 2013 D4: D 4 mg vs. Placebo
Schietroma2013 [27]	328	D 8 mg vs. Placebo, bothIV at 90****minutes before skin incision	Schietroma 2013 D8: D 8 mg vs. Placebo
Zhou2012 [28]	150	D 8 mg + T 5 mg vs. D 8 mg vs.T 5 mg, all IVimmediately before induction	Zhou 2012 Tropisetron:D 8 mg + T 5 mg vs.T 5 mg;Zhou 2012: D 8 mg vs. T 5 mg
Bononi2010 [29]	562	D 4 mg +O 4 mg vs.O 4 mg, D IV at inductionand ondansetron IV at15****minutes before tracheal extubation	Bononi 2010 Ondansetron:D 4 mg +O 4 mg vs. O 4 mg
Fujji2000 [30]	130	D 8 mg + G 40 ug/kg vs.G 40 ug/kg, both IVimmediately before induction	Fujji 2000 Granisetron:D 8 mg + G 40 ug/kg vs. G 40 ug/kg

IV: intravenous; ASA, American Society of Anesthesiologists; D: dexamethasone; T: tropisetron; O: ondansetron; G: Granisetron.

**Table 2:** Details of anesthetic technique, and rescue analgesics and anti-emetics in the included trials.

Study	Anesthetic technique	Rescue analgesics	Rescue antiemetics
Wang 1999 [18]	Propofol 2.0–2.5****mg/kg,glycopyrrolate 0.2****mg,fentanyl 2.0 ug/kg IVmaintained with 1.0%–2.5%isoflurane in oxygen	Diclofenac75****mg IM q12h	Ondansetron 4****mg IV
Wang 2000 [19]	Propofol 2.0–2.5****mg/kg,glycopyrrolate 0.2****mg,fentanyl 2.0 ug/kg IVmaintained with 1.0%–2.5%isoflurane in oxygen	Diclofenac75****mg IM q12h	Ondansetron 4****mg IV
Lee 2001 [20]	Glycopyrrolate 0.2****mg,fentanyl 2 ug/kg, thiopental5****mg/kg IV maintainedwith desflurane in oxygen	Ketorolac 15****mg IV q6h	Droperidol 1.25****mg IV
Fujji 2007 [21]	Propofol 2****mg/kg,fentanyl 2 ug/kg, vecuronium0.1****mg/kg IV maintainedwith 1–3% sevoflurane in oxygen	Indomethacin50 mg rectally	Ranitidine 150****mg orally
Worni 2008 [22]	Propofol/thiopental,atracurium, isoflurane, orsevoflurane andfentanyl 5–10 ug/kg	Acetaminophen4****g/day; second-linemetamizole1g or morphine	Ondansetron 4****mg IV; second-line droperidol 0.625****mg IV
Feroci 2010 [23]	Propofol 2****mg/kg,fentanyl 2 ug/kg,vecuronium 0.1****mg/kg IVmaintained withsevoflurane in oxygen	Paracetamol1000****mg IVq8****h;second-lineketorolac 30****mgIV q12h	Metoclopramide 10****mg IV; second-line ondansetron 4****mg IV
Doksrod 2012 [24]	Propofol, fentanyl,vecuronium IV maintainedwith desflurane (4–8%) anditrous oxide (60%) in oxygen	Oxycodone 5****mgorally; second-linemetamizole ormorphine 2.5mgIV	Metoclopramide 20****mg IV; second-line ondansetron 4****mg IV
Song 2013 [25]	Remifentanil 1 ug/kg,propofol 1–2****mg/kg,rocuroniumin 0.9****mg/kg IV,maintained with desfluranein oxygen–air mixture	Ketorolac30****mgIV	Metoclopramide 10****mg IV
Barros 2013 [26]	Propofol, fentanyl 2.0 ug/kg,cisatracurium 0.15****mg/kg,maintained withsevoflurane in oxygen	Ketorolac 30****mgorparecoxib 40****mg IV	Ondansetron 4****mg IV
Schietroma 2013 [27]	Sodium thiopental5****mg/kg,atracuriumbesylate 0.5****mg/kg,maintained with oxygenin air, sevoflurane, andremifentanil hydrochloride	Ketorolac tromethamine30****mg IV q6h	Ondansetron hydrochloride 4****mg IV
Zhou 2012 [28]	Propofol 1.5–2.5****mg/kg,midazolam 0.1–0.2****mg/kg,fentanyl 1.0–2.0 ug/kg,maintained with 1.0–3.0%sevoflurane in oxygen	Pethidine 25****mgIV	Metoclopramide 10****mg IV; second-line tropisetron 5****mg IV
Bononi 2010 [29]	Not stated^a^	Not stated	Not stated
Fujji 2000 [30]	Thiopentone 5 mg/kg,fentanyl 2 ug/kg,vecuronium0.2 mg/kg maintainedwith isoflurane (1.0%–3.0%)and nitrous oxide(66%) in oxygen	Indomethacin50mg rectally formoderae painand buprenorphiine 0.2 mg IM forsevere pain	Domperidone retally

IV, intravenous; IM, intramuscular; ^a^no difference.

**Table 3:** Quality of evidence in included studies.

Included studies	Country	Sequencegeneration	Allocationconcealment	Doubleblinding	Completeoutcome data	No selectivereporting	Baselinecomparability	Risk ofbias
Wang 1999 [18]	China	Adequate	Unclear	Yes	Yes	Yes	Yes	Low
Wang 2000 [19]	China	Adequate	Unclear	Yes	Yes	Yes	Yes	Low
Lee 2001 [20]	China	Adequate	Adequate	Yes	Yes	Yes	Yes	Low
Fujii 2007 [21]	Japan	Adequate	Adequate	Yes	Yes	Yes	Yes	Low
Worni 2008 [22]	Switzerland	Adequate	Adequate	Yes	Yes	Yes	Yes	Low
Feroci 2011 [23]	Italy	Adequate	Adequate	Yes	Yes	Yes	Yes	Low
Doksrod 2012 [24]	Norway	Adequate	Adequate	Yes	Yes	Yes	Yes	Low
Song 2013 [25]	Korea	Adequate	Adequate	Unclear	Yes	Yes	Yes	Low
Barros 2013 [26]	Portugal	Adequate	Adequate	Yes	Yes	Yes	Yes	Low
Schietroma 2013 [27]	Italy	Adequate	Adequate	Yes	Yes	Yes	Yes	Low
Zhou 2012 [28]	China	Adequate	Adequate	Unclear	Yes	Yes	Yes	Low
Bononi 2010 [29]	Italy	Adequate	Adequate	Unclear	Yes	Yes	Yes	Low
Fujii 2000 [30]	Japan	Adequate	Adequate	Yes	Yes	Yes	Yes	Low

### 3.3 Treatment effects

#### 3.3.1 Primary outcome

**Figure 2:**
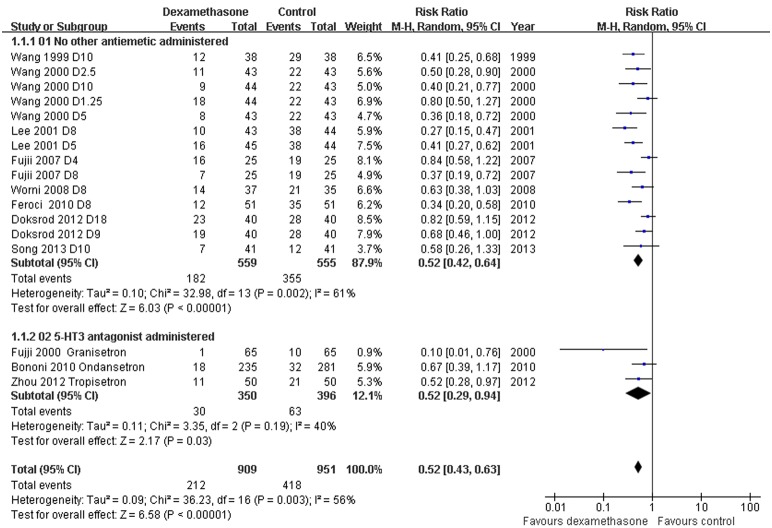
Incidence of PONV grouped by concomitant anti-emetics. Eleven studies described the incidence of PONV in thyroidectomy patients treated with dexamethasone versus placebo with or without concomitant anti-emetics (RR 0.52, 95% CI 0.43 to 0.63, *P*<0.00001). There was evidence of significant heterogeneity between studies (*P* = 0.003, *I^2^* = 56%).

**Figure 3:**
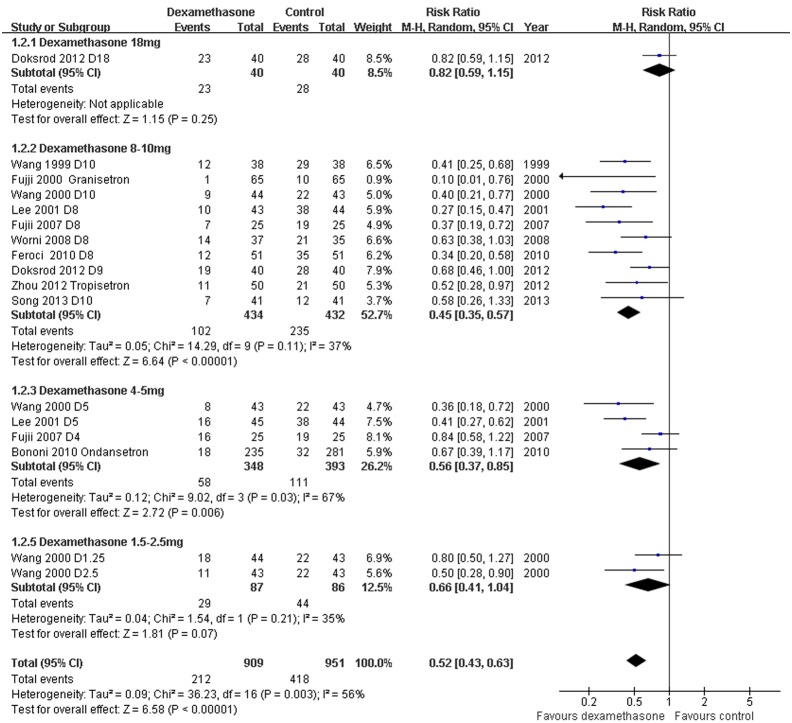
PONV according to dexamethasone dose. Higher dexamethasone doses (8–10****mg) were significantly more effective than lower dexamethasone doses (1.25–5****mg) (*P* = 0.02).

*Incidence of PONV: Dexamethasone versus placebo, with or without concomitant anti-emetics -* Data reporting on the incidence of PONV in thyroidectomy patients treated with dexamethasone versus placebo with or without concomitant anti-emetics are described in 11 RCTs [18]–[25], [28]–[30]. The meta-analysis demonstrated a significant decrease in the incidence of PONV in patients receiving dexamethasone compared to placebo, with or without concomitant anti-emetics (RR 0.52, 95% CI 0.43 to 0.63, *P*<0.00001; Fig. 2). There was evidence of significant heterogeneity between studies (*P* = 0.003, *I^2^* = 56%). The dose-response gradient may have caused most of the variation between RCTs (Fig. 3; Fig.S1).

**Figure 4:**
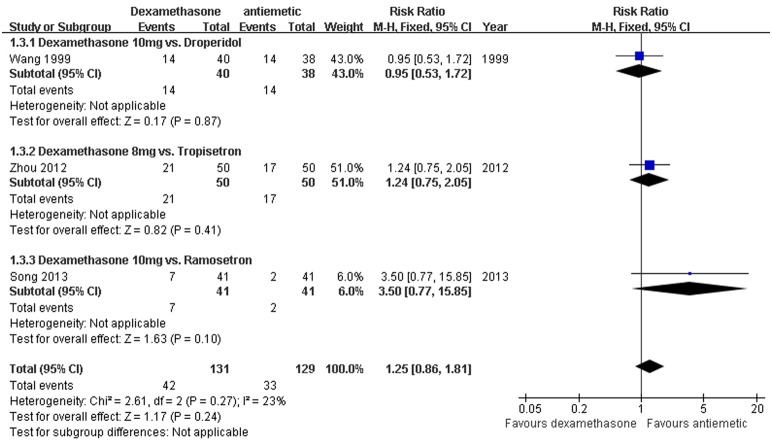
Comparison of dexamethasone with other anti-emetics. Three studies described the incidence of PONV in thyroidectomy patients treated with dexamethasone versus other anti-emetics (RR 1.25, 95% CI 0.86–1.81, *P* = 0.24). There was no evidence of significant heterogeneity between RCTs (*P* = 0.27, *I^2^* = 23%).

*Incidence of PONV: Dexamethasone versus a different antiemetic -* Data reporting on the incidence of PONV in thyroidectomy patients treated with dexamethasone versus a different anti-emetic, including droperidol, granisetron, or tropisetron, are described in three RCTs [18], [25], [28]. The meta-analysis demonstrated no significant difference in the incidence of PONV in patients receiving dexamethasone compared to these different anti-emetics (RR 1.25, 95% CI 0.86–1.81, *P* = 0.24; Fig. 4). There was no evidence of significant heterogeneity between RCTs (*P* = 0.27, *I^2^* = 23%).

#### 3.3.2 Secondary outcomes

**Figure 5:**
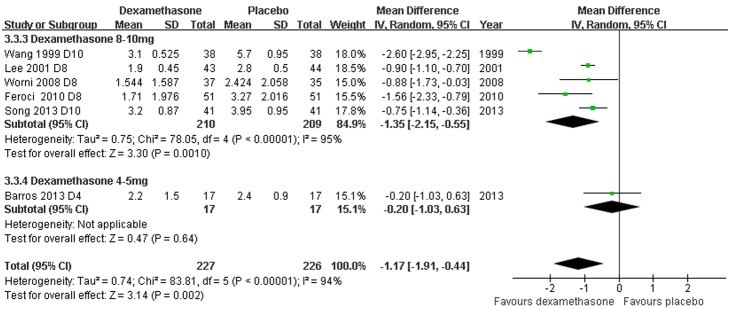
VAS post-operative pain score grouped by dexamethasone dose. Six studies described post-operative pain scores in thyroidectomy patients treated with dexamethasone versus placebo with or without concomitant anti-emetics (WMD –1.17, 95% CI –1.91 to –0.44, *P* = 0.002). There was evidence of significant heterogeneity between RCTs (*P*<0.00001, *I^2^* = 94%).

**Figure 6:**
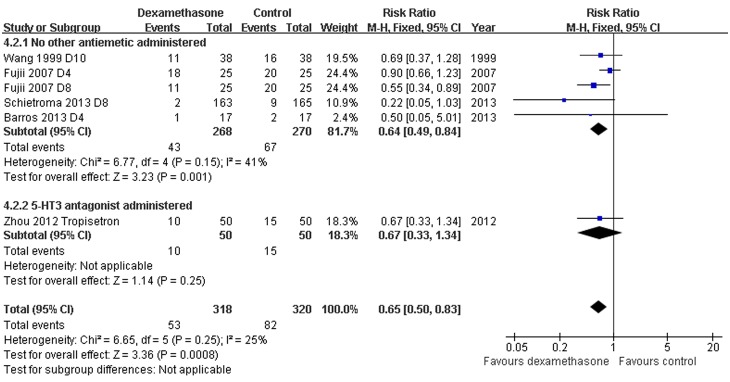
Need for rescue analgesics grouped by concomitant anti-emetics. Six studies described the need for rescue analgesics in thyroidectomy patients treated with dexamethasone versus placebo with or without concomitant anti-emetics (RR 0.65, 95% CI 0.50–0.83, *P* = 0.0008). There was no evidence of significant heterogeneity between RCTs (*P* = 0.25, *I* ^2^ = 25%).

*Postoperative pain scores and need for rescue analgesia: Dexamethasone versus placebo with or without concomitant anti-emetics* - Data reporting on post-operative pain scores in thyroidectomy patients treated with dexamethasone versus placebo with or without concomitant anti-emetics are described in six RCTs [18], [20], [22], [23], [25], [26]. Pain scores were evaluated based on visual analogue scales (VAS) completed by patients 24****h post-operatively. Four RCTs [22], [23], [25], [26] reported data as means ± standard deviations (SDs); two RCTs [18], [20] reported data as medians (range) converted to estimated means and SDs [31]. The meta-analysis demonstrated a significantly lower post-operative VAS score in patients receiving dexamethasone compared to placebo, with or without concomitant anti-emetics (WMD –1.17, 95% CI –1.91 to –0.44, *P* = 0.002; Fig. 5). There was evidence of significant heterogeneity between RCTs (*P*<0.00001, *I^2^* = 94%). The doses of dexamethasone may have caused most of the variation between RCTs. The need for rescue analgesia was significantly less frequent in the patients that received dexamethasone (RR 0.65, 95% CI 0.50–0.83, *P* = 0.0008; Fig. 6); there was no evidence of significant heterogeneity between RCTs (*P* = 0.25, *I^2^* = 25%).

**Figure 7:**
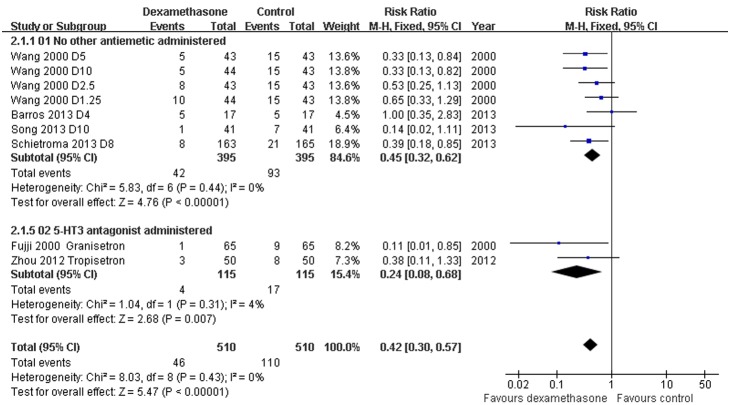
Need for rescue antiemetics grouped by concomitant antiemetics. Six studies described the need for rescue antiemetics in thyroidectomy patients treated with dexamethasone versus placebo with or without concomitant antiemetics (RR 0.42, 95% CI 0.30 to 0.57, *P*<0.00001). There was no evidence of significant heterogeneity between RCTs (*P* = 0.43, *I^2^* = 0%).

*Need for rescue anti-emetic: Dexamethasone versus placebo with or without concomitant antiemetics -* Data reporting on the need for rescue anti-emetics in thyroidectomy patients treated with dexamethasone versus placebo with or without concomitant anti-emetics are described in six studies [19], [25]–[28], [30]. The meta-analysis demonstrated a significant decrease in the need for rescue anti-emetics in patients receiving dexamethasone compared to placebo, with or without concomitant anti-emetics (RR 0.42, 95% CI 0.30 to 0.57, *P*<0.00001; Fig. 7). There was no evidence of significant heterogeneity between RCTs (*P* = 0.43, *I^2^* = 0%).

*Incidence of adverse events: Dexamethasone versus placebo with or without concomitant anti-emetics -* Data reporting on blood glucose levels in thyroidectomy patients treated with dexamethasone are described in two RCTs. A significantly higher level of blood glucose was observed in patients receiving dexamethasone compared to controls during the first 8 hours post-operatively [23], [24]. No statistical differences in symptomatic transient hypocalcemia and asymptomatic transient hypocalcemia were present [23]. One RCT [27] reported that dexamethasone administration prevented recurrent laryngeal nerve palsy; however, this effect was not described elsewhere [23]. There were no significant differences in the incidences of extrapyramidal signs including headache, dizziness, constipation, and muscle pain, and other adverse events such as wound infection and delayed wound healing, in patients receiving dexamethasone compared to controls.

### 3.4 Subgroup analyses

#### Incidence of PONV: Dose of dexamethasone

Subgroup analyses stratified by dose of dexamethasone (range, 1.25 mg to 18****mg) demonstrated that dexamethasone 4–5****mg and 8–10****mg significantly reduced the incidence of PONV compared to controls (1.25–5 mg: RR 0.59, 95% CI 0.44 to 0.79; 8–10****mg: RR 0.45, 95% CI 0.35–0.57), while dexamethasone 18****mg did not (RR 0.82, 95% CI 0.59–1.15) (Fig. 3). Dexamethasone 8–10****mg had a significantly greater effect for reducing the incidence of PONV than dexamethasone 1.25–5****mg (1.25–5****mg RR 0.40, 95% CI 0.28 to 0.55; 8–10****mg: RR 0.23, 95% CI 0.18–0.31; P = 0.02; Fig. S1).

#### Incidence of PONV: Timing of dexamethasone administration

The RCTs included in this review varied with regard to timing of dexamethasone administration. Some patients received dexamethasone 90****minutes before skin incision, while others received dexamethasone postoperatively. Wang et al [32] demonstrated that dexamethasone administered before anesthesia was more effective in decreasing early PONV compared to dexamethasone administered after anesthesia. These observations are in accordance with data showing that the onset time of dexamethasone on anti-emesis is approximately 2 hours. In the current study, subgroup analysis stratified by the timing of dexamethasone administration showed that dexamethasone was most effective in preventing PONV when administered before rather than after induction of anesthesia (*P* = 0.0002; Fig. S2).

### 3.5 Sensitivity analysis

To explore the effects of individual RCTs on the pooled OR estimates, we performed a sensitivity analysis omitting one study at a time. No single RCT significantly affected the overall results of the meta-analysis.

### 3.6 Publication bias

Visual inspection of a Funnel plot, Egger's test, and Begg's rank correlation test revealed no significant publication bias (Begg’s rank correlation test, continuity-corrected Pr>|z| >0.1), except for RCTs reporting on the need for rescue anti-emetic in patients receiving dexamethasone versus placebo, with or without concomitant antiemetics (Pr>|z|  = 0.06), (Table S2).

### 3.7 Quality of evidence

Quality of available evidence from RCTs, which was downgraded by inconsistency (heterogeneity between studies), indirectness (variations in study setting), or publication bias, and upgraded by dose-response gradient, varied from moderate to high (Table 4).

**Table 4:** GRADE evidence.

Outcomes	Illustrative comparativerisks* (95% CI)		Relativeeffect(95% CI)	No ofParticipants(studies)	Quality of theevidence(GRADE)
	Assumed risk	Corresponding risk			
	Placebo	Dexamethasone			
**Dexamethasone** **versus placebo (in** **addition to other** **antiemetics): PONV**	**440 per** **1000**	**229 per 1000**(189 to 277)	**RR 0.52**(0.43 to0.63)	1860(17 studies)	⊕⊕⊕⊕** high** 1
**Dexamethasone** **versus placebo (in** **addition to other** **antiemetics): rescue** **antiemetics**	**216 per** **1000**	**91 per 1000**(65 to 123)	**RR 0.42**(0.3 to0.57)	1020(9 studies)	⊕⊕⊕ **high**
**Dexamethasone** **comparison of doses:** **PONV**	**440 per** **1000**	**229 per 1000** (189 to 277)	**RR 0.52**(0.43 to0.63)	1860(17 studies)	⊕⊕⊕⊕** high** ^2^
**Dexamethasone versus** **placebo: VAS pain** **score**		The meandexamethasoneversus placebo:vas pain scorein the interventiongroups was**1.17 lower**(1.91 to 0.44 lower)		453(6 studies)	⊕⊕⊕⊖** moderate** ^3^
**Dexamethasone versus** **placebo (in addtion to** **other antiemetics):** **resuce analgesic**	**256 per** **1000**	**167 per 1000**(128 to 213)	**RR 0.65**(0.5 to0.83)	638(6 studies)	⊕⊕⊕⊖** moderate** ^4^
**Dexamethasone versus** **a different** **antiemetic: PONV**	**256 per** **1000**	**320 per 1000**(220 to 463)	**RR 1.25**(0.86 to1.81)	260(3 studies)	⊕⊕⊕⊕ **high**

*The basis for the **assumed risk** (e.g. the median control group risk across studies) is provided in footnotes. The **corresponding risk** (and its 95% confidence interval) is based on the assumed risk in the comparison group and the **relative effect** of the intervention (and its 95% CI). **CI:** Confidence interval; **RR:** Risk ratio.

GRADE Working Group grades of evidence. **High quality:** Further research is very unlikely to change our confidence in the estimate of effect. **Moderate quality:** Further research is likely to have an important impact on our confidence in the estimate of effect and may change the estimate. **Low quality:** Further research is very likely to have an important impact on our confidence in the estimate of effect and is likely to change the estimate. **Very low quality:** We are very uncertain about the estimate.

1 Although the PONV results demonstrated significant heterogeneity (*P* = 0.003, *I* ^2^ = 56%), it was partly explained by the dose of dexamethasone. ^2^Downgraded by not comparing higher dose with lower dose directly, but upgraded by the dose-response gradient. ^3^Although there was significant heterogeneity (*P*<0.00001, *I* ^2^ = 94%), it was partly explained by the dose of dexamethasone. ^4^Publication bias as *Pr*>|*z*| = 0.06.

PONV: post-operative nausea and vomiting; VAS: visual analogue scales.

**Patient or population:** patients undergoing thyroidectomy. **Settings:** evidence from China, Japan, Korea, Italy, Switzerland, Norway, Portugal. **Intervention:** dexamethasone. **Comparison:** placebo.

## Discussion

PONV is a common and distressing complication for patients undergoing thyroidectomy; therefore, prophylactic anti-emetics may be beneficial. An optimal anti-emetic regimen should be capable of decreasing the incidence of PONV without increasing the risk for adverse events. However, most of the currently used anti-emetics, including anti-histamines, butyrophenones, and dopamine receptor antagonists cause occasional undesirable adverse events, such as excessive sedation, hypotension, dry mouth, dysphoria, hallucinations, and extrapyramidal signs [33]. 5-HT3 antagonists are effective for preventing and treating PONV in patients undergoing various types of surgery [34]. However, the use of prophylactic anti-emetic therapy with 5-HT3 antagonists has been criticized for being too expensive [35].

Our meta-analysis of 13 RCTs demonstrated that prophylactic dexamethasone is effective in reducing the incidence of PONV, post-operative pain scores, and the need for rescue analgesia and anti-emetics compared to placebo administered with or without contaminant anti-emetics in patients undergoing thyroidectomy. In addition, our findings showed that dexamethasone is as effective as other anti-emetics for reducing PONV in this patient population. However, the benefits of administering dexamethasone as a more cost-effective anti-emetic and efficacious analgesic drug^35^ should be weighed against the potential side effects. Our study indicated that dexamethasone administration is associated with an increase in blood glucose during the immediate post-operative period, but with no other serious adverse events.

For optimal dose and timing of dexamethasone administration, subgroup analyses showed that higher doses of dexamethasone (8–10****mg) are more effective than lower doses (1.5–5****mg), and dexamethasone is most effective in preventing PONV when administered before rather than after induction of anesthesia.

In terms of populations eligible for treatment, the RCTs in the current study mostly included healthy patients, and excluded pregnant women, patients with insulin-dependent diabetes mellitus, those who were obese, and patients with a high risk for PONV. As such, the impact of prophylactic dexamethasone on outcomes in high-risk patients is not known. A larger sample size and well-performed RCTs including high risk patients are required for further investigations.

This review has several limitations. First, data reporting on the effects of prophylactic dexamethasone on post-operative pain scores and need for rescue analgesics in thyroidectomy patients were limited by substantial heterogeneity and publication bias, respectively. Second, the studies in this systematic review included patients across various age groups receiving dexamethasone according to very different protocols.

## Conclusion

The present meta-analysis shows that prophylactic dexamethasone is safe and effective for reducing the incidence of PONV, post-operative pain, and the need for rescue analgesia and anti-emetics in thyroidectomy patients. Prophylactic dexamethasone 8–10****mg administered before induction of anesthesia should be recommended for patients undergoing thyroidectomy except for those that are pregnant, have diabetes mellitus, hyperglycaemia or contraindications for corticosteroids. More high quality trials are warranted to define the benefits and risks of prophylactic dexamethasone in potential patients with high risk for PONV.

## Supporting Information

Figure S1 **Incidence of PONV stratified according to dexamethasone dose: 8–10 mg and 1.25–5 mg.**
(TIF)

Figure S2 **Incidence of PONV stratified by timing of dexamethasone administration.**
(TIF)

Table S1 **Search strategy from its inception to October 1, 2013.**
(DOC)

Table S2 **Begg’s rank correlation test for publication bias.**
(DOC)

Checklist S1 **PRISMA 2009 Checklist.**
(DOC)
