# Supplementary figures and images for: The Impact of Prophylactic Dexamethasone on Nausea and Vomiting after Thyroidectomy: A Systematic Review and Meta-Analysis
Source: PLoS One. 2014 Oct 16;9(10):e109582. doi: 10.1371/journal.pone.0109582 (PMC4199613; doi:10.1371/journal.pone.0109582)

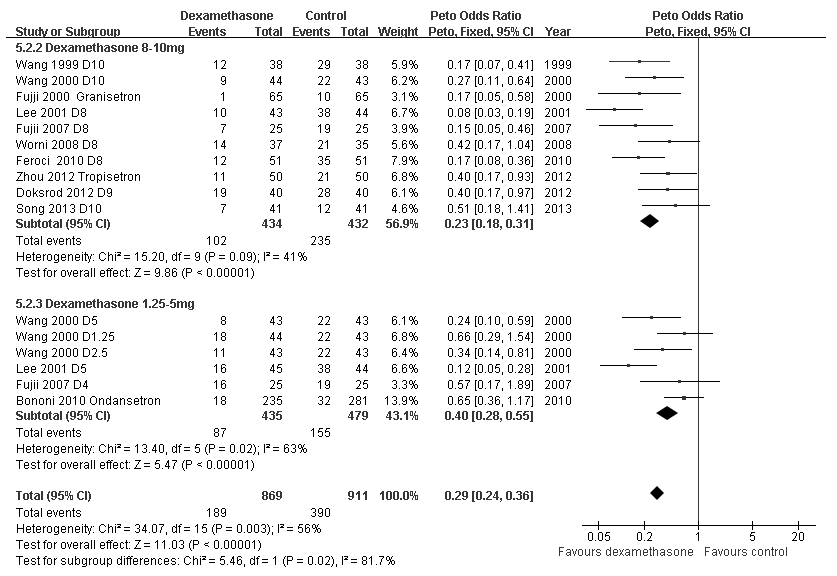

Supplement: Figure S1 — Incidence of PONV stratified according to dexamethasone dose: 8–10 mg and 1.25–5 mg. (TIF) [file pone.0109582.s001.tif]

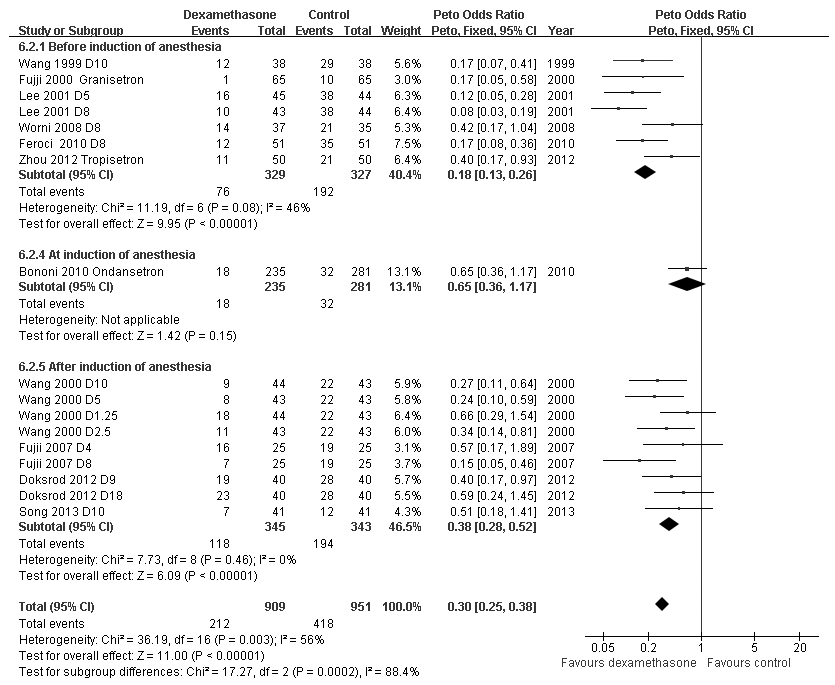

Supplement: Figure S2 — Incidence of PONV stratified by timing of dexamethasone administration. (TIF) [file pone.0109582.s002.tif]
