# Supplementary material for: The Impact of Prophylactic Dexamethasone on Nausea and Vomiting after Thyroidectomy: A Systematic Review and Meta-Analysis
Source: PLoS One. 2014 Oct 16;9(10):e109582. doi: 10.1371/journal.pone.0109582 (PMC4199613; doi:10.1371/journal.pone.0109582)
Supplement: Table S1 — Search strategy from its inception to October 1, 2013. (DOC) [file pone.0109582.s003.doc]

Table S1 Search strategy from its inception to October 1, 2013

| 1 | randomized controlled trial [pt] |
| --- | --- |
| 2 | controlled clinical trial [pt] |
| 3 | randomized [tiab] |
| 4 | placebo [tiab] |
| 5 | drug therapy [sh] |
| 6 | randomly [tiab] |
| 7 | trial [tiab] |
| 8 | groups [tiab] |
| 9 | #1 or #2 or #3 or #4 or #5 or #6 or #7 or #8 |
| 10 | Dexamethasone [tiab] |
| 11 | steroid [tiab] |
| 12 | glucocorticoid [tiab] |
| 13 | #10 or #11 or #12 |
| 14 | thyroid surgery [tiab] |
| 15 | thyroidectomy [tiab] |
| 16 | thyroidectomy [Mesh] |
| 17 | #14 or #15 or #16 |
| 18 | #9 and #13 and #17 |
