# Supplementary material for: The Impact of Prophylactic Dexamethasone on Nausea and Vomiting after Thyroidectomy: A Systematic Review and Meta-Analysis
Source: PLoS One. 2014 Oct 16;9(10):e109582. doi: 10.1371/journal.pone.0109582 (PMC4199613; doi:10.1371/journal.pone.0109582)
Supplement: Table S2 — Begg’s rank correlation test for publication bias. (DOC) [file pone.0109582.s004.doc]

Table S2 Begg’s rank correlation test for publication bias

| Variable | *Pr*>|*z*| |
| --- | --- |
| Dexamethasone versus placebo (in addition to other antiemetics): PONV | 0.303 |
| Dexamethasone versus placebo (in addition to other antiemetics): rescue antiemetic | 0.175 |
| Dexamethasone comparison of doses: PONV | 0.303 |
| Dexamethasone versus placebo (in addition to other antiemetics): pain | 0.707 |
| Dexamethasone versus placebo (in addition to other antiemetics): rescue analgesic | **0.06^a^** |
| Dexamethasone versus a different antiemetic: PONV | 0.296 |

^a^ Publication bias existed as *Pr*>|*z*| value was ≤0.1.
